# Editorial: Community series in immune responses against tumors - from the bench to the bedside, volume II

**DOI:** 10.3389/fimmu.2025.1719075

**Published:** 2025-10-17

**Authors:** Chun Jing Wang, Shisan Bao

**Affiliations:** ^1^ Institute of Immunity & Transplantation, University College London Division of Infection & Immunity, London, United Kingdom; ^2^ Scientific Research Division, The Third Affiliated Hospital, Gansu University of Chinese Medicine, Baiyin, Gansu, China

**Keywords:** tumour, bench to bed, immune response, editorial, community

Cancer immunology has become a cornerstone of modern oncology, providing opportunities for early detection, precise prognosis, and innovative treatment. This second volume of the *Community Series in Immune Responses Against Tumours – From the Bench to the Bedside* presents a curated collection of original research, reviews, and case studies that collectively expand our understanding of tumour immunology. The contributions cover biomarker development, immune cell dynamics, mechanistic exploration, emerging therapeutic avenues, and vaccine-based strategies, underscoring how molecular insights are increasingly integrated with clinical practice. Collectively, these studies demonstrate the movement of immune-based discoveries toward meaningful therapeutic impact.

## Biomarkers and diagnostic strategies

Reliable biomarkers continue to be pivotal in cancer management. Zhou et al. analysed serum total immunoglobulin E (IgE) levels and lung cancer risk in a retrospective cohort of 675 patients and 1,193 healthy controls. Patients with lung cancer had significantly elevated IgE levels, with 47.9% above 100 IU/ml, which was associated with more advanced tumour stages, although progression-free and overall survival were not significantly different. This study identifies IgE as a possible diagnostic marker and draws attention to allergic immune pathways as therapeutic opportunities, particularly in older patients with smoking histories and altered monocyte counts.

In a complementary study, Zhou et al. examined a panel of seven tumour-associated autoantibodies (7-TAABs) in oesophageal squamous cell carcinoma (ESCC). The combined 7-TAAB assay improved sensitivity and diagnostic accuracy compared with single-antibody tests and was associated with clinical features such as tumour location, size, and TNM stage. These findings highlight how multi-antibody panels could support earlier detection and refined clinical risk assessment in ESCC.

## Tumour immune microenvironment and mechanistic insights

A detailed characterisation of the tumour immune microenvironment (TIME) is essential for progress in immunotherapy. Guo et al. reviewed T cell subsets in cervical cancer, emphasising their functional diversity, spatial distribution, and interactions with other immune cells. Variations in T cell subsets across histological subtypes and disease stages influence anti-tumour responses and affect outcomes of therapies such as immune checkpoint blockade and HPV-directed vaccination. De Miranda et al. showed that local tumour ablation can trigger systemic immune effects, with cryoablation of primary breast cancers inducing an abscopal effect on distant lesions. These results suggest that combining local interventions with systemic immunomodulation can enhance anti-tumour responses, offering a promising route for combined therapeutic strategies.

Adding molecular depth, Zeng et al. investigated the CXCR7–TAGLN2 protein complex in papillary thyroid carcinoma (PTC), using an integrated approach that combined clinical tissue analysis, *in vitro* studies, and mechanistic experiments. Immunohistochemistry of 64 PTC and 24 benign thyroid tissues demonstrated markedly elevated CXCR7 and TAGLN2 expression, both of which were significantly linked to lymph node metastasis and positively correlated with each other. Co-localisation and co-immunoprecipitation assays confirmed their physical interaction. Functionally, silencing TAGLN2 suppressed PTC cell migration, while CXCR7 overexpression reversed this effect. Mechanistic studies revealed that TAGLN2 knockdown reduced phosphorylated Smad2 (p-Smad2) levels, implicating TAGLN2 in TGF-β/Smad2 pathway activity, while re-introduction of CXCR7 restored p-Smad2 expression. Together, these findings indicate that CXCR7 promotes invasion and metastasis through TAGLN2-mediated activation of TGF-β/Smad2 signalling. Identification of the CXCR7–TAGLN2 complex as a regulator of metastatic progression highlights a potential therapeutic target in PTC.

## Case reports and clinical innovations

Managing rare or complex cancers frequently requires personalised and multimodal approaches. Zeng et al. described a patient with synchronous lung adenocarcinoma and oesophageal squamous cell carcinoma who achieved survival beyond three years following chemotherapy, definitive chemoradiotherapy, stereotactic body radiation therapy (SBRT), and anti-PD-1 immunotherapy. This case illustrates the feasibility of integrating systemic and local therapies in patients with multiple primary malignancies while maintaining acceptable safety.

Similarly, Xiong et al. reported a patient with advanced pulmonary large-cell neuroendocrine carcinoma (LCNEC) who received first-line chemotherapy followed by sovantinib and toripalimab. The patient achieved a partial response and a progression-free survival of 15.1 months, highlighting the promise of combining immune checkpoint inhibition with targeted agents in rare and aggressive cancers. Both cases reinforce the importance of tailoring therapeutic strategies to tumour biology and immune context.

## Cancer vaccines and immunoprevention

Vaccination approaches represent a growing frontier in cancer prevention and treatment. Asadollahi et al. designed a multi-neoepitope vaccine (MNEV) against non-small cell lung cancer (NSCLC) using reverse vaccinology and bioinformatics. In murine models, MNEV elicited robust B and T cell responses, enhanced IFN-γ and granzyme B secretion, and increased CD4+ and CD8+ T cell populations, providing support for the further development of neoepitope-based vaccines in personalised immunotherapy.

Zhang et al. identified a Ddx21 mutant peptide (Ddx21MT) as an effective neoantigen that elicited strong anti-tumour immunity in mice. Vaccination expanded central memory T cell populations and provided long-term protection, underscoring its potential in prophylactic lung cancer vaccination. Taken together, these investigations show how computational design coupled with experimental validation can generate next-generation cancer vaccines with translational promise.

## Conclusion

The studies in this volume reflect the breadth and depth of contemporary cancer immunology. From biomarker identification to mechanistic investigations, and from immune microenvironment analysis to innovative therapies and vaccines, these contributions demonstrate how molecular, cellular, and clinical perspectives converge to advance patient care. Mechanistic studies such as the CXCR7–TAGLN2 investigation show how dissecting specific molecular interactions can guide the rational development of targeted therapies and immune-based strategies. By linking molecular targets with immune-focused interventions and vaccine approaches, these works move experimental discoveries closer to clinical application and advance the goals of precision oncology.

We thank all contributing authors and reviewers whose work has shaped this volume into a timely and valuable reference for researchers and clinicians committed to understanding and leveraging immune responses against tumours.

This is also supported by a grant from The Gansu Provincial Science and Technology Project (Project No 25RCKD001).

